# Suicide in Bangladesh: An Ecological Systems Analysis

**DOI:** 10.1002/brb3.70233

**Published:** 2024-12-31

**Authors:** S. M. Yasir Arafat, Tamkeen Saleem

**Affiliations:** ^1^ Department of Psychiatry Bangladesh Specialized Hospital Dhaka Bangladesh; ^2^ Biomedical Research Foundation Dhaka Bangladesh; ^3^ Department of Psychology International Islamic University Islamabad Pakistan

**Keywords:** ecology of suicide, prevention, risk factors, socioecological model, suicide in Bangladesh

## Abstract

**Background:**

Suicide happens due to a complex interaction among multiple factors. Hence, prevention strategies should consider a wider range of personal and ecological aspects. It is an underprioritized public health problem in Bangladesh where risk factors for suicide have been poorly studied, and there is no national suicide prevention strategy. Therefore, exploring socioecological aspects of suicidal behavior would foster suicide prevention in the country.

**Objectives:**

We aim to discuss the ecological model for suicide and suicide prevention in Bangladesh.

**Methods:**

We performed a narrative review and organized the risk factors and prevention strategies according to the socioecological model in the context of Bangladesh. We used ecological systems to analyze risk factors for suicide and suicide prevention strategies in the country.

**Results:**

Based on the available evidence, we categorized the risk factors for suicide into ontogenic system, microsystem, mesosystem, exosystem, macrosystem, and chronosystem. In addition, we contextualized the existing suicide prevention initiatives and potential suicide prevention strategies in ontogenic system, microsystem, mesosystem, exosystem, macrosystem, and chronosystem.

**Conclusions:**

This article explored suicide and suicide prevention in Bangladesh through the lens of ecological systems theory that may foster the formulation of a national suicide prevention strategy in the country.

## Graphical abstract

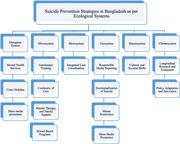

## 1 Introduction

### 1.1 Suicide in Global Perspective

Suicide is a global problem affecting family, country, and society (Cerel et al. 2016). About 726,000 suicides happen in a year in the world (World Health Organization 2024). Over the past 45 years, there has been a 65% increase in the global suicide rate (World Health Organization 2014). Suicide ranks as the leading cause of death across all ages; however, among young people aged 15–29 years, it ranks as the third most common cause of death (World Health Organization 2024). For each suicide, there are about 20 more attempts indicating the hidden burden (Mohammadnezhad, Konrote, and Kabir 2018). In addition, about six persons are bereaved by each suicide, and exposure to suicide is associated with an individual's suicidal behavior, post‐traumatic stress disorder, depressive disorder, and prolonged grief (Cerel et al. 2016). Suicide is a complex outcome among multiple factors ranging from genetic factors, environmental factors, personal factors, social factors, and cultural factors (Zalsman et al. 2016; Greig and Kabir 2022). Identification of definite risk factors with the definite association between suicide attempts and risk factors is yet to be revealed (World Health Organization 2014; Zalsman et al. 2016).

### 1.2 Suicide in Bangladesh

In Bangladesh, suicide has been a significant public health concern, but unfortunately, it has somewhat failed to receive the prioritization it warrants as a major public health issue (Arafat 2019). Bangladesh lacks a national suicide surveillance system, and to date, there has been no comprehensive nationwide research conducted to investigate suicidal risk factors (Arafat 2024a). In addition, suicide remains classified as a criminal offense within the legal system (Arafat 2017). Religious and social factors have a strong influence on all domains of life in Bangladesh, and thus these continue to influence the stigma, diagnosis registration, and laws of suicides. Furthermore, the families also refrain from disclosing the true nature of the death/act due to fear of police harassment, social stigma, and religious aspects (Bose, Kushal, and Arafat 2023).

Factors contributing to suicide in Bangladesh include social and economic issues such as poverty, unemployment, domestic violence, and stigma associated with mental health issues (Arafat, Mohit, et al. 2021; Gibson and Hossain 2017). Several researchers have examined individual, family, peer, and school‐level risk factors for suicide in Bangladesh (Arafat, Mohit, et al. 2021; Arafat, Saleem, et al. 2022; Feroz et al. 2012; Reza et al. 2014; Tasfiand Mostofa 2024; Sharmin Salam et al. 2017). However, risk factors of wider variables like legal status of suicide attempt, provision of adequate clinical services, inadequate budgetary allocation, role of media, health economics aspects and burden of suicide attempt, self‐harm, and poisoning have not yet been researched.

Most of the studies have, however, overlooked broader perspectives of suicide and suicide prevention considering the socioecological models, such as cultural representations, beliefs and values, religious practice and values, social justice, economic freedom, enduring gender roles, marital and family systems, and law‐ and policy‐level changes, which are inherently pertinent. For a better understanding of suicidality in Bangladesh, it is crucial to examine all levels of the wider socioecological variables. People live in interconnected systems that originate from their immediate environment and extend to the broader societal environment.

### 1.3 Ecological Systems Theory and Socioecological Model

**FIGURE 1:**
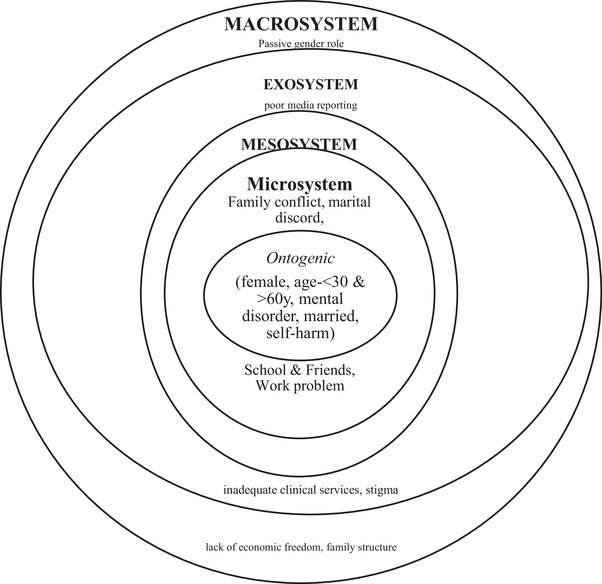
Risk factors for suicide in Bangladesh in different ecological systems.

According to the ecological systems theory developed by Bronfenbrenner (1977, 1979, 1977, 1979), individuals exist within interconnected systems, such as family and school, which extend to broader environmental contexts like neighborhood and culture, influencing individual attitudes and behaviors. The ecological systems theory postulates that individuals can be situated within microsystem, mesosystem, exosystem, macrosystem, and chronosystem levels (Figure 1, for systems). At the center, there is an ontogenic system that comprises individual factors (Ayyash‐Abdo 2002). The microsystem surrounds the ontogenic system immediately that comprises family members, the school environment, religious establishments, friends, and health services (Henderson 2020). Mesosystem is recognized as a “system of microsystems.” It is shaped by different dimensions of relationships among the microsystems. Social systems make the exosystem. It comprises neighbors, social supports, mass media, political milieu, and the structure of the economy. The next circle is the macrosystem, which comprises enduring cultural norms, ethics, beliefs, and standards. The chronosystem is made up of enduring environmental and climate changes.

The social‐ecological model (SEM) of suicide acknowledges the intricate interplay between sociocultural and environmental factors and their impact on an individual's suicidal behavior (Centers for Disease Control and Prevention 2024; Cramer and Kapusta 2017; Zulkiply and Rosliza 2022). This model emphasizes the multifaceted nature of human behavior, recognizing that individuals are influenced by various levels of their environment, including interpersonal relationships, community settings, societal norms, and broader cultural contexts that influence suicidality. Therefore, while considering suicide prevention, these factors are needed to be considered. This model started with an individual's suicidal behavior interacting with the microsystem, mesosystem, exosystem, and macrosystem (Henderson 2020). By considering these interconnected layers, the SEM provides a comprehensive framework for understanding and addressing complex social and health issues, such as suicidal behavior, by identifying and targeting factors at multiple levels of influence.

### 1.4 The Current Study

Suicide happens due to a complex interaction among multiple factors. Hence, prevention strategies should consider a wider range of personal and ecological risk factors. Suicidality is an underprioritized public health problem in Bangladesh where risk factors for suicide have been poorly studied, and there is no national suicide prevention strategy. Therefore, exploring socioecological aspects of suicidal behavior would foster the development of a national suicide prevention strategy in the country. Based on this background, we aim to discuss suicide in Bangladesh considering the socioecological model and contextualizing the ecological systems theory.

## 2 Methods

### 2.1 Study Design

We performed a narrative review and organized the available evidence in regard to the ecological systems theory for suicide and suicide prevention in Bangladesh.

### 2.2 Search Strategy

We searched PubMed, Scopus, Google, BanglaJOL, and Google Scholar to identify the articles mentioning risk factors for suicide and suicide prevention. We used “suicide in Bangladesh” as the search term. We accessed all articles published in the English language from inception to March 2024. In addition, we conducted a hand search of review articles and a recently published book “*Suicide in Bangladesh*” (Arafat and Khan 2023).

### 2.3 Screening and Categorizing

**FIGURE 2:**
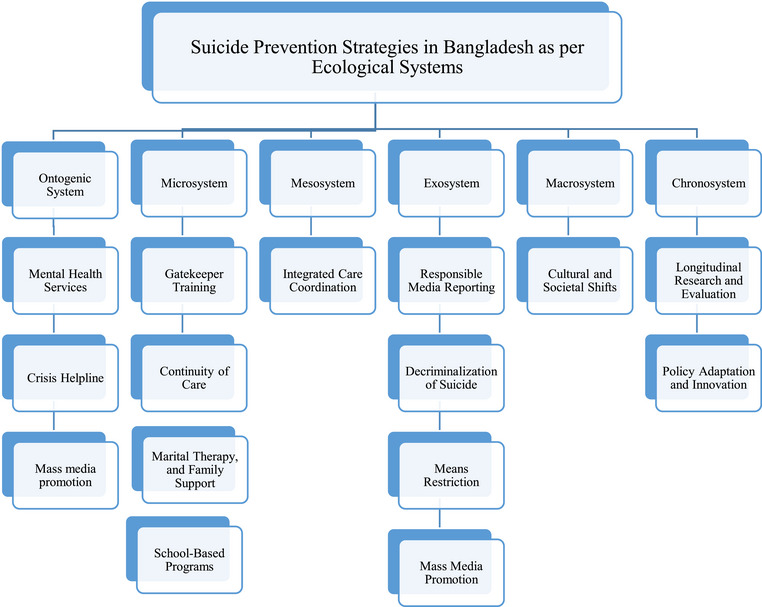
Proposed suicide prevention strategies in Bangladesh considering the ecological systems.

We included any article discussing risk factors for suicide and suicide prevention in Bangladesh context, published in English language, and available in full texts. We excluded articles published in other languages, articles discussing other countries, and articles without full text. Initially, we assessed the titles and abstracts of the articles and then we checked the full text in Microsoft Excel Software. In this narrative review, we categorized the variables according to the ecological systems. Therefore, we did not perform any statistical analysis. We categorized the risk factors for suicide (Figure 1) and suicide prevention strategies (Figure 2) according to the ecological systems (ontogenic system, microsystem, mesosystem, exosystem, macrosystem, and chronosystem).

### 2.4 Ethical Approval

We did not seek any formal clearance from any institutional review board as we reviewed already published papers.

## 3 Risk Factors for Suicide in Bangladesh in Different Ecological Systems

Risk factors for suicide in Bangladesh emerge from complex interactions across multilayered ecological contexts. We categorized the risk factors for suicide in different ecological systems (Figure 1).

### 3.1 The Individual Factors/Ontogenic System

The research literature highlights various individual‐level risk factors associated with suicide in Bangladesh. Among them, psychiatric disorders including personality disorder and substance abuse, unemployment, married status, female gender, adverse life events, and previous history of self‐harm attempts are significant (Arafat 2023; Arafat, Mohit, et al. 2021; Feroz et al. 2012; Reza et al. 2014; Arafat 2019). One systematic review identified that the majority of the suicides happened among persons with age under 30 years (Arafat 2019) whereas one community‐based study identified that the highest rate was observed in the 60+ age group, with considerably high rates also reported among adolescents (Mashreky, Rahman, and Rahman 2013). A study reported that unskilled laborers were 16 times more likely to attempt suicide than students indicating the role of personal factors in suicidality (Sharmin Salam et al. 2017). Sometimes, there are multiple individual‐level risk factors in an individual and there are interactions among individual factors and with other systems. For instance, a person with a major psychiatric disorder may lose the job and become unemployed.

### 3.2 Microsystem

It comprises family members, the school environment, religious establishments, friends, and health services (Henderson 2020). Risk factors related to family and friends, schools and religious institutions, and health services pertain to the system. Interpersonal problems, marital issues, domestic or intimate partner abuse, complex gender role expectations, and the cultural norm of extended family structures are considered significant contributors to suicidal behavior in South Asian culture, including Bangladesh (Khan 2002). The data from a case‐control psychological autopsy study indicate that about 86% of suicides could be prevented if life events are prevented (Arafat, Khan, et al. 2021). Shah, Ahmed, and Arafat (2017) revealed family conflicts, cheating in relations, extramarital affairs, and domestic violence as risk factors for suicide attempts. A systematic review of the literature revealed relational factors such as experiences of sexual abuse, involvement in extramarital relationships, instances of child marriage, the loss of a partner and/or children, domestic violence, and divorce as potential risk factors for suicide in the country. Among these factors, marital discord and family conflict emerged as primary contributors to suicide (Arafat 2019). Sibling conflicts, issues in joint family business, and nonsupportive or strained relationships among the siblings were revealed as risk factors within the family system (Arafat, Saleem, et al. 2022). A psychological autopsy study showed that 40% of the life events were closely linked to interpersonal violence and associated with marital and sexual issues. These included spousal discord, extramarital affairs, forced marriages, premarital love relationships, and conflicts with family members (Arafat, Mohit, et al. 2021).

Academic stress or crisis, exam failure, bullying or ragging, break up of relations, and lack of peer support or peer relational issues can also elevate the risk of suicidal behavior in schools. Many of these factors are inter‐related. For instance, first‐year students, in particular, often fall victim to bullying and ragging, which negatively impacts their academic performance and mental health, and thus it all may contribute to suicidal behavior (Urme et al. 2022). Another study reported that infrequent parental supervision and insufficient peer support, loneliness, experiencing bullying, and lacking close friends were associated with a higher likelihood of suicidal behavior in schools in the country (Khan et al. 2020).

### 3.3 Mesosystem

A mesosystem comprises the interrelationships between two or more microsystems in which the child is involved, such as the relationship between family and school. Experiences in one microsystem, such as the home environment, may influence activities and interventions in another, such as peer relationships in school, and vice versa (Eamon 2001). For instance, unemployment, poverty, relationship problems, drug addiction, political marginalization, and the stigma and shame are all connected and can cause low self‐esteem and suicidal thoughts (Tasfi and Mostafa 2024). It has been noticed that suicide happens due to the demand unfulfillment by young kids and being scolded or bitten by teachers (Shah, Ahmed, and Arafat 2017; Arafat 2019). Disharmony with parents, disapproval of marriage after a premarital love affair, and suicidal behavior in response to the problems with mother‐in‐laws have been identified in the country (Shah, Ahmed, and Arafat 2017; Arafat 2019).

### 3.4 Exosystem

In Bangladesh, suicide is considered as a criminal offense. Criminal legal status hinders the health‐seeking behavior, increases stigma, and under‐reporting of suicidal behavior (Bose, Kushal, and Arafat 2023).

Social problems such as social isolation, stigma related to seeking help, and lack of counseling centers are identified as significant factors for suicide (Arafat, Hussain, et al. 2022; Shahnaz et al. 2017). In Bangladeshi society, taboos and stigmas surrounding mental health illnesses prevail, leading to feelings of hopelessness among students and acting as barriers to seeking treatment for mental health issues (Urme et al. 2022).

Bangladesh, predominantly a Muslim country, not only holds strong religious prohibitions against suicide and suicidal attempts but also enforces punitive laws against attempted suicide (Bose, Kushal, and Arafat 2023). Religious, cultural, and social factors, along with legal repercussions, often deter individuals from disclosing suicidal thoughts or attempts.

There are inadequate clinical services and trained human resources in Bangladesh. There are only two suicide prevention clinics in the country with irregular functioning (Arafat 2024a).

The quality of suicide reporting by media can have both positive and negative impacts on suicidal behavior among the masses. The quality of suicide reporting in Bangladesh varies among print media, online news platforms, and motion pictures. Media professionals barely follow World Health Organization (WHO) guidelines for reporting suicides (Anik 2023). A study reported that harmful media reporting practices were prevalent, with detailed suicide methods reported in 75.5% of newspaper articles. Conversely, potentially helpful reporting practices were almost nonexistent, as no articles provided contact details for a suicide support service (Arafat, Mali, and Akter 2019). Research is needed to provide recommendations for the development and implementation of guidelines for media reporting quality and the development of the national suicide prevention policy.

### 3.5 Macrosystem

In Bangladesh, just like in other South Asian cultures, complex gender role expectations and extended family structures are considered contributing factors to suicidal behavior (Khan 2002). Bangladeshi families at times face conflicts when parents try to keep their children under the norms of their culture and extended family (Ghuman 2000). The role of the extended family can have both positive and negative impacts. Arguments with nonresident family members that may be extended family members and issues in the combined family business may fuel suicidal behaviors (Arafat, Mohit, et al. 2021; Arafat 2019). However, the extended family can also buffer the suicide‐related thoughts or behaviors. The research suggests that industrialization and urbanization have led to social change in the structure of families as becoming more nuclear and focused on work. In Bangladesh, this has resulted in a lack of communication and support between parents and their children, leaving younger generations feeling lonely and unsupported during times of crisis. Previously, members of extended family used to provide care and support but new family dynamics have limited that (Tasfi and Mostafa 2024).

### 3.6 Chronosystem

Bangladesh is a disaster prone country that is experiencing rising temperature and humidity and deforestation due to urbanization. Climate change and experiencing disasters affect mental health and suicidality (Wahid, Islam, and Raza 2024).

## 4 Suicide Prevention in Bangladesh in Different Ecological Systems

Considering the risk factors for suicide in Bangladesh, in this section, we propose prevention strategies fitting with the ecological systems at multiple levels, including individual, microsystem, mesosystem, and exosystem levels (Figure 2).

### 4.1 The Individual‐Level Interventions

#### 4.1.1 Mental Health Services

Mental illness is an important risk factor for suicide. The rate of psychiatric morbidity in suicide was 61% (Arafat, Mohit, et al. 2021). Treatment of psychiatric disorders is an important suicide prevention strategy (Zalsman et al. 2016). Therefore, proper and enduring treatment of mental illness should be prioritized. Currently, there is no available specialized mental health service in Bangladesh for persons with suicidal behavior. Previously, there were two suicide prevention clinics that have been ceased recently (Arafat 2024b). Policy‐level attention is warranted to ensure adequate services for persons with suicidal behavior extending to the community level.

#### 4.1.2 Crisis Helplines

Interpersonal relationship problems and sexual abuse are important domains of risk factors for suicide in Bangladesh (Arafat 2019). Crisis intervention would potentially prevent suicide attempts (WHO 2014). “Kaan Pete Roi” is an important helpline for suicide prevention working for more than 10 years (Arafat 2024a). However, the helpline is not available on a 24/7 basis and the helpline number is not unique. “999” is the governmental helpline for emergencies that has been playing a role in suicide prevention. There is a strong need to establish hotlines to manage suicidal behaviors where the staffed personnel should be trained counselors who may provide immediate support and intervention for individuals in crisis.

#### 4.1.3 Mass Media Promotion

Educative mass media promotion should be targeted to raise awareness about suicide prevention and the availability of suicide prevention services so that individual people can avail when necessary (Zalsman et al. 2016; World Health Organization 2023).

### 4.2 Microsystem‐Level Interventions

#### 4.2.1 Gatekeeper Training

It is a proven suicide prevention strategy where family members, teachers, peers, physicians, and religious leaders have access to the community people and can identify as individual with suicidal behavior (Kingi‐Uluave et al. 2024; Zalsman et al. 2016, WHO 2014). The strategy showed its impact on suicide prevention in many settings among different populations (Kingi‐Uluave et al. 2024). Application of this preventive measure is yet to be started in Bangladesh. It needs to train the gatekeepers so that they can identify the risky individuals and refer them to the potential preventive services.

#### 4.2.2 Continuity of Care

Family members play vital roles in the continuity of mental health care after discharge from the hospital for an acute episode and also in long‐term follow‐up in the treatment of chronic psychiatric disorders (Arafat, Saleem, et al. 2022; Edwards, Patterson, and Griffith 2021).

#### 4.2.3 Marital Therapy and Family Support

Conflicts with spouses, extramarital relationships, premarital affairs, and marital discords are the prominent immediate life events of suicide in Bangladesh. Therefore, access to family counseling services and the establishment of support groups can help to improve communication, strengthen relationships, and provide caregivers with strategies to support loved ones at risk of suicide (Edwards, Patterson, and Griffith 2021; Ayyash‐Abdo 2002).

#### 4.2.4 School‐Based Programs

Implementation of suicide prevention programs in schools that teach students and educators about mental health, coping skills, and how to support peers in distress. Peers can be a significant source of identification and support for mental health difficulties to identify the red flags for suicide and assist in help‐seeking behavior. There should be training programs for the staff and teachers of the educational institutes. Teachers and school staff should be trained to recognize warning signs of suicide and should intervene appropriately. Inclusion of content on mental health and suicide literacy would be beneficial in the long term.

### 4.3 Mesosystem‐Level Interventions

#### 4.3.1 Integrated Care Coordination

Facilitation of communication and collaboration between health‐care providers, schools, workplaces, neighborhood, and community organizations and mental health services to ensure coordinated care for individuals at risk of suicide. There is a need to develop referral pathways and protocols for a seamless transition between different levels of care, such as primary care, mental health clinics, and crisis services. The schools, workplaces, community organizations, and mental health‐care facilities should have liaisons for integrated care coordination.

### 4.4 Exosystem‐Level Interventions

#### 4.4.1 Responsible Media Reporting

The quality of media reporting of suicide is grossly irresponsible (Anik 2023). Although some initiatives have been ensured like training the journalist by WHO and the National Institute of Mental Health (NIMH), Dhaka, there is no visible impact of it. Public health attention is warranted to improve the quality of media reporting. Conducting seminars, workshops, and awareness‐raising campaigns is imperative to address the quality of media reporting of suicidal behavior in Bangladesh.

From September to November 2018, the WHO and the NIMH conducted workshops for journalists on responsible reporting of suicide. Ten media houses, including print, electronic, and TV channels, were invited to attend. The workshops were based on the WHO kit “Preventing Suicide: A Resource for Media Professionals, Update 2017,” which offered guidance on “Dos” and “Don'ts” to ensure safe and responsible media coverage of suicides. These workshops were part of a broader WHO initiative aimed at promoting mental health and well‐being and enhancing mental health service delivery (WHO Bangladesh 2018, 2022, 2018, 2022; Anik 2023).

#### 4.4.2 Decriminalization of Suicide

Suicide is a criminal offense in Bangladesh that hampers help‐seeking behavior. Decriminalization would be a major step for suicide prevention in the country (Arafat 2019).

#### 4.4.3 Means Restriction

Bangladesh banned Category I pesticides from agriculture in 2000 that reduced suicides by poisons (Chowdhury et al. 2018).

#### 4.4.4 Mass Media Promotion

Regular promotions in mass media targeting stigma reduction and increasing suicide literacy would be considered. It will raise awareness among general people regarding the risky signs of suicidal persons, available services for suicide prevention, and myths about suicide.

### 4.5 Macrosystem‐Level Interventions

#### 4.5.1 Cultural and Societal Shifts

With the change in the social fabric in Bangladesh, due to nuclear family living and career orientation, there is a strong need to promote cultural and societal shifts that prioritize mental health and well‐being, including fostering supportive communities, promoting connectedness, and encouraging help‐seeking behaviors. Ensuring human rights and gender equity in every sector would ease the difficulties faced by females, especially at an early age.

### 4.6 Chronosystem‐Level Interventions

To understand the impact of climate on suicide‐related behaviors and its determinants in Bangladesh, it is imperative to conduct longitudinal research to track changes in suicide rates, risk factors, and protective factors over time. Use data to evaluate the effectiveness of prevention and intervention programs and inform future strategies. Continuously adapting and innovating suicide prevention policies and interventions in response to changing societal dynamics, emerging technologies, and new evidence‐based practices are warranted. Foster collaboration between government agencies, NGOs, academic institutions, and other stakeholders to drive innovation and knowledge sharing in suicide prevention.

## 5 Strength and Limitations

In this paper, we aimed to contextualize ecological systems theory in Bangladesh explaining the risk factors and prevention initiatives of suicide. However, there are several limitations. First, it is a commentary type of narrative review to explain an emerging concept in suicidology conceptually without adequate empirical evidence suggesting the model fit. Therefore, there may be personal biases while considering the risk factors and prevention initiatives. Second, as a commentary type of narrative review, we did not follow systematic documentation of search strategies. Also, we extracted the risk factors and prevention initiatives from the same search. Third, it is important to consider that suicide is multifactorial with a complex interaction among the factors. Therefore, there may be overlap between the systems.

## 6 Conclusion

Understanding the ecological systems or socioecological factors is essential for developing effective national suicide prevention strategies that are sensitive to the evolving sociocultural and economic context of Bangladesh. This conceptual article explored suicide in Bangladesh through the lens of ecological systems theory that may foster the formulation of a national suicide prevention strategy and the prioritization of resource utilization based on the ecological model of suicide prevention. However, this conceptual paper warrants empirical testing to determine the associations among the variables with suicidal behavior. Longitudinal community‐based studies are warranted to measure the direction and strength of associations. Also, the associations of suicidal behavior with ecological variables are complex due to multiple confounders and mediators.

## Author Contributions

**S. M. Yasir Arafat**: conceptualization, methodology, investigation, validation, writing–original draft, writing–review and editing, project administration, supervision. **Tamkeen Saleem**: writing–original draft, writing–review and editing.

## Conflicts of Interest

The authors declare no conflicts of interest.

### Peer Review

The peer review history for this article is available at https://publons.com/publon/10.1002/brb3.70233.

## Data Availability

Data sharing is not applicable to this article as no new data were created or analyzed in this study.
